# Dr. Verlegh’s Case of Amaurosis Cured by Inoculation with the Sulphate of Strychnia

**Published:** 1844-09-21

**Authors:** 


DR. VERLEGH'S CASE OF AMAUROSIS CURED BY INOCULATION WITH THE SULPHATE OF STRYCHNIA.

The subject was a lady, 27 years of age, of nervous temperament, affected with incomplete amaurosis of the left eye, and commencement of the same disease in the right one. The disease was of three months standing, and of rheumatic origin; after two months' fruitless efforts, Dr. Verlegh tried inoculation with the sulphate of strychnia in the neighbourhood of the orbit. A grain of the salt was dissolved in two drops of water; the first day 12 inoculations were performed, six above the eye in the course of the supra-orbital nerve, six under, and on the side of the nose where the ethmoidal filaments and nasal branch terminate, and whence arise the filaments which go to the iris. There was no effect that day; but next day some slight tremors occurred in the neighbourhood of the inoculated spots. After two days' rest the inoculations were repeated, and the number of punctures increased to 18. The patient now became sensible of a slight haziness. After five successive inoculations carried to the length of 30 punctures, she commenced to distinguish objects; after the eighth, vision was completely restored; the contraction of the pupil gradually increased, and the other symptoms diminished after five grains of the sulphate had been used; during the same time inoculations were had recourse to in the neighbourhood of the right eye; after the lapse of two months the patient continued perfectly restored; and this the author conceived sufficiently long to warrant him in considering the cure as permanent.—*Ibid. from ibid.*

